# Enhancing Pediatric Patient Safety by Evaluating the Effect of Targeted Education on Nurses’ Knowledge, Attitudes, and Practices of Fall Prevention

**DOI:** 10.7759/cureus.82946

**Published:** 2025-04-24

**Authors:** Naseema Shafqat, Tanya Rawat, Vishakha Singh, Ranjana Verma, Manisha Rani, Sunil K Tailor

**Affiliations:** 1 Obstetrics and Gynecology, All India Institute of Medical Sciences, Bhopal, Bhopal, IND; 2 Pediatrics, All India Institute of Medical Sciences, Bhopal, Bhopal, IND; 3 Medical Surgical Nursing, All India Institute of Medical Sciences, Bhopal, Bhopal, IND

**Keywords:** attitudes, fall prevention, healthcare quality improvement, kap, knowledge, patient safety, pediatric care, pediatric nursing, practices

## Abstract

Background

Patient safety in pediatric care is a critical concern, as hospital environments pose unique risks for children, particularly falls. Despite advancements in healthcare, falls remain a common safety issue, emphasizing the need for effective preventive measures. Nurses play a vital role in reducing fall risks, but gaps in their knowledge, attitudes, and practices (KAP) can hinder the success of these efforts. This study evaluates the impact of targeted education on enhancing nursing officers' KAP of fall prevention.

Materials and methods

A quasi-experimental pre-test and post-test design was conducted in the pediatric units of a tertiary care hospital in Bhopal. Nursing officers with at least one year of pediatric experience participated. KAP was assessed before a structured educational intervention, which included a workshop and educational materials. One week post-intervention, data were collected to measure changes. Statistical analysis was performed using SPSS Statistics version 26 (IBM Corp. Released 2019. IBM SPSS Statistics for Windows, Version 26.0. Armonk, NY: IBM Corp.), with paired t-tests assessing improvements in KAP and chi-square tests examining associations with demographic variables.

Results

A total of 64 nurses participated, with the majority being between 26 and 30 years old and holding a Bachelor of Science in Nursing degree. Pre-intervention knowledge levels indicated that most participants had average knowledge, which significantly improved post-intervention (mean score: 16.2 ± 4.20 to 24.0 ± 3.37, p<0.001). Attitudes toward fall prevention became more positive (mean score: 69.7 ± 6.07 to 73.5 ± 4.82, p<0.001), and preventive practices improved significantly (mean score: 10.7 ± 2.15 to 12.8 ± 1.50, p<0.001). Knowledge was significantly associated with age (p=0.029) and marital status (p=0.021), while attitudes and practices were linked to the department and the age group of the children in care.

Conclusions

Targeted educational interventions significantly improved nurses' KAP of pediatric fall prevention. Although the intervention led to immediate improvements, further research is needed to assess long-term retention and sustainability. Continuous professional development, institutional policies, and interdisciplinary collaboration are essential to maintaining high standards of pediatric patient safety.

## Introduction

Patient safety is a fundamental aspect of healthcare, aiming to prevent avoidable incidents that may harm patients. A key factor contributing to such incidents is a lack of situational awareness, which increases the risk of adverse outcomes [1]. Pediatric patients are particularly vulnerable due to the unique challenges of the hospital environment. Research has emphasized the need for comprehensive fall prevention strategies that extend beyond clinical staff directly caring for patients. While staff training is essential, it is often not specifically tailored to nurses, leading to gaps in care quality [2].

The World Health Organization (WHO) defines a fall as "an event that results in a person coming to rest inadvertently on the ground or floor or other lower level" [3]. Falls are measured by the number of incidents per 1,000 patient days and serve as a key nursing-sensitive quality indicator in inpatient care. Falls are a leading cause of safety incidents among hospitalized children. In 2016, a patient safety report documented 5,562 fall incidents, including 254 involving children, with falls accounting for 24% of pediatric safety events [4]. Additionally, falls rank as the 12th leading cause of death in children aged 5-9 years and 15-19 years [5]. The WHO highlights that pediatric patients are at increased risk due to their developmental stage and mobility patterns. Despite advances in healthcare, studies suggest that up to 30% of hospitalized pediatric patients experience falls during admission [3].

Nurses play a crucial role in fall prevention as frontline caregivers. However, gaps in knowledge, attitudes, and practices (KAP) can hinder the effectiveness of fall prevention interventions. Research indicates that targeted education for nursing officers enhances awareness and promotes evidence-based fall prevention strategies, resulting in reduced fall rates. Well-structured educational programs can equip nurses with the necessary skills to identify high-risk patients, apply preventive measures, and foster a culture of safety in pediatric care settings [6].

This quality improvement study aims to assess the impact of targeted education on nurses' KAP of pediatric fall prevention. The findings will contribute to existing research and inform practical strategies to strengthen patient safety initiatives in healthcare settings.

## Materials and methods

Study design, setting, participants, and sample selection

This quasi-experimental study employed a one-group pre-test and post-test design and was conducted in the pediatric units of a tertiary care center in Bhopal, India. The study included nurses who provided direct care in pediatric units. Participants were selected using purposive sampling to ensure the inclusion of those actively involved in pediatric patient care. The inclusion criteria required nurses to have at least one year of experience in pediatric care and to provide written informed consent. Nurses were excluded if they had recently completed fall prevention training (within the last six months), were on extended leave, or were unavailable for post-intervention assessment. The estimated sample size was 55 participants, calculated using OpenEpi software (Dean AG, Sullivan KM, Soe MM. OpenEpi: Open Source Epidemiologic Statistics for Public Health. www.OpenEpi.com) at a 5% level of significance and 80% power, based on the expected improvement in knowledge scores as the primary outcome measure. Out of 80 nurses screened for eligibility, 64 provided consent, completed both the pre-test and post-test assessments, and were included in the final analysis.

Data collection instruments

A structured, pre-validated questionnaire was used to assess nursing officers' KAP of pediatric fall prevention. The tool was developed after an extensive review of relevant literature and clinical guidelines. A panel of experts in pediatric nursing and patient safety reviewed it for content validity. Section I focused on the demographic and professional characteristics of the participants and included 14 items, such as age, gender, educational qualification, total years of nursing experience, and specific expertise in pediatric care. Section II assessed knowledge related to fall risks, prevention strategies, and safety protocols using thirty multiple-choice questions. Section III measured attitudes toward fall prevention through 5-point Likert-scale statements to evaluate participants' beliefs and perceptions. This section included 18 Likert-scale items that explored participants' beliefs and perceptions about fall prevention, such as their sense of responsibility ("I would feel guilty if a child fell during my shift") and confidence in their ability to prevent falls ("I believe I have the necessary skills to prevent falls"), as well as views on the role of parents and the hospital environment ("I believe that falls occur because of the parents’ irresponsible behavior", "The hospital environment is safest; therefore, chances of falling are less"). Section IV assessed fall prevention practices using a 15-item observation checklist to evaluate the nursing officers' adherence to safety measures during routine pediatric care. The same questionnaire was used in both pre- and post-intervention assessments to evaluate the effectiveness of the educational workshop.

Ethical considerations

The study was approved by the Institutional Human Ethics Committee of the All India Institute of Medical Sciences, Bhopal (approval number: IHEC/SR/2024/88, approval date: February 1, 2024) and adhered to ethical research guidelines. Written informed consent was obtained from all participants before enrollment, and strict confidentiality was maintained throughout the study.

Data collection procedure and intervention

The study was conducted in two phases: in Phase I (baseline assessment), participants completed a structured KAP questionnaire to assess their existing KAP of pediatric fall prevention. Demographic and professional details were also collected to analyze potential associations with KAP scores. In Phase II (educational intervention), a hands-on workshop was conducted in small groups of four over two consecutive days, with 32 participants attending each day to ensure effective interaction and individual attention. Each workshop session lasted for around three hours and included interactive lectures, demonstrations, case-based discussions, and simulation exercises on fall prevention strategies. Participants received informational brochures and handouts to reinforce learning. A feedback session was also held, allowing participants to clarify any doubts and enhance their understanding. Post-intervention data were collected one week later using the same structured KAP questionnaire to measure changes in KAP.

Statistical analysis

Data were analyzed using SPSS Statistics version 26 (IBM Corp. Released 2019. IBM SPSS Statistics for Windows, Version 26.0. Armonk, NY: IBM Corp.). The normality of continuous variables was assessed using the Shapiro-Wilk test (p-values: 0.088, 0.301, 0.205), confirming the suitability of parametric tests. Descriptive statistics, including mean, standard deviation, frequency, and percentage, were used to summarize sociodemographic and clinical variables. The paired t-test was conducted to compare pre-test and post-test KAP scores, assessing the effectiveness of the intervention. The chi-square test examined associations between demographic variables and KAP scores.

## Results

Participant characteristics

Table 1 summarizes the participants' characteristics. Most nursing officers (59.37%) were between the ages of 26 and 30. Female nurses comprised 54.68% of the workforce, while male nurses accounted for 45.31%. A majority (79.68%) held a Bachelor of Science in Nursing degree, and most (84.37%) were nursing officers, with 14.06% being senior nursing officers. Over half (53.12%) were married, while 46.87% were unmarried. Among them, 29.68% had children, while 70.31% did not. Half (50%) had one to three years of experience, and the largest group (34.37%) worked in the pediatric medicine ward. Most (90.62%) worked rotating shifts, and 89.06% had never witnessed a fall incident. Only 26.56% had received formal training on fall prevention, but 67.18% were aware of existing protocols. Nearly all (96.87%) believed fall prevention practices were essential in pediatric units.

**Table 1:** Sociodemographic characteristics of participants (N=64) BSc: Bachelor of Science, MSc: Master of Science, PICU: pediatric intensive care unit, SMTU: severe malnutrition treatment unit, PSICU: pediatric surgical intensive care unit

S. No.	Sociodemographic variables		Frequency	Percentage (%)
1.	Age	21-25	10	15.62
		26-30	38	59.37
		31-35	15	23.43
		>35	1	1.56
2.	Gender	Male	29	45.31
		Female	35	54.68
3.	Educational qualification	Diploma in nursing and midwifery	3	4.68
		Post basic BSc nursing	4	6.25
		BSc nursing	51	79.68
		MSc nursing	6	9.37
		Others	0	0
4.	Designation	Nursing officer	54	84.37
		Senior nursing officer	9	14.06
		Assistant nursing superintendent	1	1.56
5.	Marital status	Unmarried	30	46.87
		Married	34	53.12
		Divorced/separated	0	0
		Widow/widower	0	0
6.	Do you have kids	Yes	19	29.68
		No	45	70.31
7.	Work experience	1-3 years	32	50
		3-5 years	11	17.18
		>5 years	21	32.81
8.	Department	Pediatric medicine ward	22	34.37
		Pediatric surgery	12	18.75
		PICU	15	23.43
		SMTU	11	17.18
		Pediatric emergency	3	4.68
		PSICU	1	1.56
9.	Shift schedule	General shift	6	9.37
		Rotating shift	58	90.62
10.	Have you witnessed any fall incidents at your workplace?	No	57	89.06
		Yes	7	10.93
11.	Have you received any training on fall prevention?	Yes	17	26.56
		No	47	73.43
12.	Are there any existing protocols for fall prevention within the hospital?	Yes	43	67.18
		No	21	32.81
13.	Your posting at the pediatric unit was by	Choice/as per request	6	9.37
		Obligated by employer	54	84.37
		Based on specialty	4	6.25
14.	Do you think fall prevention practices are necessary in the pediatric unit?	Yes	62	96.87
		No	1	1.56
		Maybe	1	1.56

Nurses' KAP pre- and post-intervention

Before the intervention, 76.6% of participants had average knowledge of fall prevention, with a mean score of 16.2 ± 4.20 SD, whereas only 14.06% had good knowledge and 9.37% had poor knowledge. Post-intervention, 71.9% demonstrated good knowledge, with a mean score of 24.0 ± 3.37 SD. The mean knowledge score increased significantly from 16.2 in the pre-test to 24.0 in the post-test, demonstrating a notable enhancement in participants' understanding of fall prevention. The reduction in SD from 4.20 to 3.37 suggests a more uniform improvement in knowledge across participants.

Attitudes toward fall prevention were already positive in 96.9% of participants before the intervention, and this increased to 98.4% afterward. Mean scores improved from 69.7 ± 6.07 SD to 73.5 ± 4.82 SD, indicating a stronger recognition of the importance of fall prevention. The decrease in SD from 6.07 to 4.82 reflects greater consistency in attitudes post-intervention, suggesting a more uniform commitment to fall prevention strategies.

A significant improvement was also observed in participants' fall prevention practices, with 81.3% demonstrating good practices before the intervention and 90.6% afterward, as mean scores increased from 10.7 ± 2.15 SD to 12.8 ± 1.50 SD. The reduction in SD from 2.15 to 1.50 indicates that participants not only improved their practices but also applied them more consistently.

The educational intervention significantly improved participants' knowledge (p<0.001), attitudes (p<0.001), and practices (p<0.001) related to fall prevention. A reduction in score variability indicated a more consistent improvement across participants (Table 2).

**Table 2:** Pre-test and Post-test comparison of nurses' KAP of pediatric fall prevention * p-values were generated using Student’s t-test KAP: knowledge, attitudes, and practices, SD: standard deviation

		Mean	SD	Statistic t-value	df	p-value*
Knowledge	Pre-test	16.2	4.20	-14.006	63	<0.001
	Post-test	24.0	3.37			
Attitudes	Pre-test	69.7	6.07	-10.366	63	<0.001
	Post-test	73.5	4.82			
Practices	Pre-test	10.7	2.15	-10.123	63	<0.001
	Post-test	12.8	1.50			

These findings highlight the effectiveness of the targeted educational intervention in enhancing KAP of fall prevention, with statistically significant improvements across all measures.

Associations between KAP and sociodemographic and clinical factors

Knowledge levels were significantly associated with age (p=0.029) and marital status (p=0.021). Participants aged 26-30 demonstrated better knowledge. A higher proportion of unmarried participants were categorized in the "poor" knowledge group compared to married participants, suggesting lower knowledge levels among the unmarried group. Attitudes were significantly influenced by department (p=0.001), with a higher proportion of staff in the pediatric medicine ward exhibiting more positive attitudes toward fall prevention compared to staff in other departments. No significant associations were found between knowledge and gender (p=0.131), educational qualification (p=0.269), work experience (p=0.147), prior training (p=0.145), or fall prevention protocols (p=0.637). Additionally, knowledge was not affected by factors such as having children (p=0.493), the number or age of children (p=0.198 and p=0.176, respectively), or shift schedule (p=0.630). For fall prevention practices, a significant association was found with the age of the participants' children (p=0.025), with those caring for toddlers being more likely to adopt better practices. No significant associations were observed with gender, work experience, or prior training (Tables 3-5).

**Table 3:** Association between knowledge and sociodemographic and clinical factors † indicate significant association BSc: Bachelor of Science, MSc: Master of Science, PICU: pediatric intensive care unit, SMTU: severe malnutrition treatment unit, PSICU: pediatric surgery intensive care unit

Variable		Level of knowledge			^χ²^	df	p-value
		Poor	Average	Good			
Age	21-25 years	1	9	0	14.073	6	0.029^†^
	26-30 years	2	33	3			
	31-35 years	2	10	3			
	>35 years	1	0	0			
Gender	Male	5	21	3	4.063	2	0.131
	Female	1	31	3			
Educational qualification	Diploma in nursing and midwifery	0	3	0	7.596	6	0.269
	Post basic BSc nursing	1	3	0			
	BSc nursing	3	43	5			
	MSc nursing	2	3	1			
Designation	Nursing officer	3	46	5	7.445	4	0.114
	Senior nursing officer	3	5	1			
	Assistant nursing superintendent	0	1	0			
Marital status	Unmarried	3	31	0	7.703	2	0.021^†^
	Married	3	21	6			
Have kids	Yes	2	14	3	1.415	2	0.493
	No	4	38	3			
No. of kids	1	1	13	2	3.242	2	0.198
	2	1	1	1			
Age of the kids	Infant	1	3	1	8.952	6	0.176
	Toddler	0	8	1			
	Preschooler	0	3	0			
	Schooler	1	0	1			
Work experience	1-3 years	1	29	2	6.799	4	0.147
	3-5 years	3	7	1			
	>5 years	2	16	3			
Department	Pediatric medicine ward	2	18	2	16.726	10	0.081
	Pediatric surgery ward	3	8	1			
	PICU	1	12	2			
	SMTU	0	11	0			
	Pediatric emergency	0	3	0			
	PSICU	0	0	1			
Shift schedule	General	1	4	1	0.924	2	0.630
	Rotating	5	48	5			
Witnessed any fall incidents at the workplace	No	5	47	5	0.498	2	0.780
	Yes	1	5	1			
Received training on fall prevention	Yes	3	14	0	3.863	2	0.145
	No	3	38	6			
Are there any existing protocols for fall prevention within the hospital?	Yes	4	36	3	0.903	2	0.637
	No	2	16	3			
Posting to the pediatric unit	By choice/upon request	2	4	1	5.736	4	0.220
	Mandated by employer	3	45	5			
	Based on specialty	1	3	0			
Fall prevention practices are necessary at pediatric units	Yes	6	50	6	0.476	4	0.976
	No	0	1	0			
	Maybe	0	1	0			

**Table 4:** Association between attitudes and sociodemographic and clinical factors † indicate significant association BSc: Bachelor of Science, MSc: Master of Science, PICU: pediatric intensive care unit, SMTU: severe malnutrition treatment unit, PSICU: pediatric surgery intensive care unit

Variable		Attitude		^χ²^	df	p-value
		Poor	Good			
Age	21-25 years	0	10	0.695	3	0.874
	26-30 years	1	37			
	31-35 years	0	15			
	>35 years	0	1			
Gender	Male	0	29	0.842	1	0.359
	Female	1	34			
Educational qualification	Diploma in nursing and midwifery	0	3	0.259	3	0.968
	Post basic BSc nursing	0	4			
	BSc nursing	1	50			
	MSc nursing	0	6			
Designation	Nursing officer	1	53	0.188	2	0.910
	Senior nursing officer	0	9			
	Assistant nursing superintendent	0	1			
Marital status	Unmarried	1	33	0.896	1	0.344
	Married	0	30			
Have kids	Yes	0	19	0.429	1	0.513
	No	1	44			
No. of kids	1	0	16	Attitude is constant		
	2	0	3			
Age of the kids	Infant	0	5	Attitude is constant		
	Toddler	0	9			
	Preschooler	0	3			
	Schooler	0	2			
Work experience	1-3 years	1	31	1.016	2	0.602
	3-5 years	0	11			
	>5 years	0	21			
Department	Pediatric medicine ward	0	22	20.656	5	0.001^†^
	Pediatric surgery ward	0	12			
	PICU	0	15			
	SMTU	0	11			
	Pediatric emergency	1	2			
	PSICU	0	1			
Shift schedule	General	0	6	0.105	1	0.746
	Rotating	1	57			
Witnessed any fall incidents at the workplace	No	1	56	0.125	1	0.724
	Yes	0	7			
Received training on fall prevention	Yes	0	17	0.367	1	0.544
	No	1	46			
Are there any existing protocols for fall prevention within the hospital?	Yes	0	43	2.080	1	0.149
	No	1	20			
Posting to the pediatric unit	By choice/upon request	0	7	0.211	2	0.900
	Mandated by employer	1	52			
	Based on specialty	0	4			
Fall prevention practices are necessary at pediatric units	Yes	1	61	0.033	2	0.984
	No	0	1			
	Maybe	0	1			

**Table 5:** Association between practices and sociodemographic and clinical factors † indicate significant association BSc: Bachelor of Science, MSc: Master of Science, PICU: pediatric intensive care unit, SMTU: severe malnutrition treatment unit, PSICU: pediatric surgery intensive care unit

Variable		Practices			^χ²^	df	p-value
		Poor	Average	Good			
Age	21-25 years	0	4	6	3.182	3	0.364
	26-30 years	0	19	19			
	31-35 years	0	4	11			
	>35 years	0	0	1			
Gender	Male	0	12	17	0.014	1	0.905
	Female	0	15	20			
Educational qualification	Diploma in nursing and midwifery	0	1	2	2.679	3	0.444
	Post basic BSc nursing	0	1	3			
	BSc nursing	0	24	27			
	MSc nursing	0	1	5			
Designation	Nursing officer	0	25	29	2.574	2	0.276
	Senior nursing officer	0	2	7			
	Assistant nursing superintendent	0	0	1			
Marital status	Unmarried	0	13	21	0.465	1	0.496
	Married	0	14	16			
Have kids	Yes	0	8	11	0.00	1	0.993
	No	0	19	26			
No. of kids	1	0	8	8	2.591	1	0.107
	2	0	0	3			
Age of the kids	Infant	0	1	4	9.337	3	0.025^†^
	Toddler	0	7	2			
	Preschooler	0	0	3			
	Schooler	0	0	2			
Work experience	1-3 years	0	14	18	3.703	2	0.157
	3-5 years	0	7	4			
	>5 years	0	6	15			
Department	Pediatric medicine ward	0	10	12	9.456	5	0.092
	Pediatric surgery ward	0	2	10			
	PICU	0	5	10			
	SMTU	0	8	3			
	Pediatric emergency	0	2	1			
	PSICU	0	0	1			
Shift schedule	General	0	2	4	0.213	1	0.645
	Rotating	0	25	33			
Witnessed any fall incidents at the workplace	No	0	24	33	0.001	1	0.970
	Yes	0	3	4			
Received training on fall prevention	Yes	0	7	10	0.010	1	0.992
	No	0	20	27			
Are there any existing protocols for fall prevention within the hospital?	Yes	0	20	23	1.005	1	0.316
	No	0	7	14			
Posting to the pediatric unit	By choice/upon request	0	1	6	4.032	2	0.133
	Mandated by the employer	0	23	30			
	Based on specialty	0	3	1			
Fall prevention practices are necessary at pediatric units	Yes	0	25	37	2.829	2	0.243
	No	0	1	0			
	Maybe	0	1	0			

## Discussion

This study highlights the significant impact of a targeted educational intervention on improving nurses' KAP of fall prevention. The results indicate substantial improvements across all three domains, reinforcing the crucial role of education in strengthening fall prevention measures within healthcare settings.

The findings of this study align with previous research regarding the distribution of gender, age, and experience among participants. For example, a cross-sectional study found that over half of the nurses were female, with more than two-thirds aged between 20 and 30 years, and the majority being unmarried. Additionally, a significant proportion of nurses had less than five years of experience and were employed as staff nurses [7]. A common trend across various studies is the predominance of female nurses in the workforce, particularly in pediatric nursing, where female staff constitute over 50% [8,9].

Regarding educational qualifications, most participants in this study held a bachelor's degree in nursing, which is consistent with findings from an Egyptian survey that reported a high prevalence of bachelor's degrees among ICU nurses [8]. The emphasis on fall prevention in pediatric care is supported by other studies, which have shown that most nurses recognize the importance of fall prevention protocols, indicating a shared commitment to patient safety across healthcare settings [10]. However, a cross-sectional study found that most nurses were aged 31-40, with 59.9% having received undergraduate training in fall prevention, which contrasts with our findings [11].

This study reported a lower incidence of falls (10.93%) compared to other studies, which documented rates of 21% and 78.4% [12,13]. Furthermore, 84.37% of nurses in this study were assigned to the pediatric unit by their employers. In contrast, previous research suggests that a greater proportion of nurses choose their pediatric specialization based on personal interest [14]. These differences may be attributed to variations in recruitment policies and cultural factors across institutions and countries. The findings offer valuable insights into workforce composition and training experiences, which are essential for optimizing workforce planning, training programs, and patient safety in pediatric care.

Hospital falls are largely preventable, and nurses with sufficient knowledge of fall risk factors are better equipped to reduce the occurrence of falls and improve outcomes for hospitalized pediatric patients. This study found a significant improvement in nurses' knowledge following the intervention, accompanied by a reduction in standard deviation, indicating greater consistency in knowledge levels. These results align with prior research, which demonstrates that structured educational programs enhance healthcare providers' understanding of safety protocols and evidence-based practices [9,12,13]. Additionally, a study in Saudi Arabia found a significant relationship between nurses' knowledge and factors such as nationality and work experience, as well as between attitudes and age [11].

Although this study demonstrated immediate improvements in knowledge, previous research indicates that knowledge retention may decline over time without continuous education [15]. In contrast, an Egyptian study identified significant knowledge gaps, particularly in high-risk areas such as fall prevention [16]. Other studies have reported similar deficiencies in pediatric safety knowledge, particularly regarding child safety policies and fall prevention [17,18]. Notably, our findings contradict a study that found no significant difference in knowledge among nurses with prior education on falls [13]. These discrepancies may result from variations in the type, intensity, or duration of educational interventions.

A significant association was found between knowledge levels and age, with participants aged 26-30 demonstrating higher levels of knowledge. Additionally, marital status was linked to knowledge, with married nurses showing greater knowledge than unmarried ones. However, no significant associations were found between knowledge and factors such as gender, educational qualification, work experience, department, or prior training. These results align with previous studies that highlight associations between knowledge, age, and marital status. However, findings regarding other demographic factors have been inconsistent [11,12,16]. Contrarily, some studies reported no significant associations between knowledge and demographic variables [9].

These findings are largely consistent with existing literature, demonstrating the positive impact of educational interventions on nurses' knowledge of fall prevention in pediatric care. However, notable discrepancies remain, particularly regarding the sustainability of knowledge over time and variations in baseline knowledge levels among nurses from different regions. These contrasting findings highlight the complexities of knowledge acquisition in nursing and the varying influences of demographic factors, underscoring the need for further research to explore these differences. Additionally, the study emphasizes the necessity for continuous professional development to uphold high standards of care.

The positive shift in attitudes observed in this study, reflected in a mean score increase from 69.7 to 73.5, indicates a greater recognition of the importance of fall prevention. This change suggests a more proactive approach to patient safety, essential for fostering a culture of safety in healthcare settings. The findings align with studies conducted in Tamil Nadu, Manipur (India), and South Korea, which have found that educational interventions improve attitudes toward patient safety [12,15,19]. Similarly, a study in Shanghai, China, reported improvements in both attitudes and behaviors related to fall prevention following educational interventions [7]. Some studies have noted that pre-existing positive attitudes are linked to nurses' knowledge and professional development [11,13]. However, not all studies reported improvements in attitudes post-intervention, with some suggesting that factors such as the intervention content, duration, and contextual challenges within healthcare settings may influence attitude changes [20-22].

In this study, attitudes toward fall prevention varied by departmental affiliation, suggesting that the nature of care and patient population may influence perspectives on fall prevention. No significant relationships were found between attitudes and age, gender, education, marital status, work experience, or prior training. These results are consistent with studies that show attitudes were influenced by departmental affiliation, but not necessarily by demographic factors [11,16]. Further research is needed to explore these associations in greater detail and better understand the factors that influence nurses' attitudes toward fall prevention.

Improvements in fall prevention practices following the educational intervention indicate that the acquired knowledge and positive attitude changes were effectively applied in clinical settings. Nurses demonstrated a greater commitment to preventive behaviors, such as conducting thorough risk assessments and implementing safety measures, post-intervention. These findings are consistent with previous studies that report improvements in fall prevention practices post educational interventions [11,23,24].

However, discrepancies exist in the literature, with some studies finding that two-thirds of nurses displayed inadequate practices related to child safety [20]. Another study reported that while educational interventions improved knowledge, they did not lead to significant changes in practice [21,25]. These differences suggest that contextual factors, such as the healthcare environment, managerial support, and intervention design, may influence the translation of knowledge into practice. Continuous education and a supportive work environment are essential to ensuring sustained improvements in clinical practice. Additionally, this study found a significant association between the age of participants' children and fall prevention practices, with nurses caring for infants demonstrating greater awareness of fall prevention strategies. However, no significant associations were found between fall prevention practices and age, gender, education, work experience, or prior training. These findings contradict studies that found associations between fall prevention practices and nurses' age and educational backgrounds [11,16].

This study focuses on pediatric nurses and their role in fall prevention, addressing the unique needs of children. While much of the research on fall prevention has focused on adults, pediatric fall prevention strategies require specialized approaches due to the developmental and physiological differences in children [26,27]. This study provides valuable insights into enhancing safety practices in pediatric care and underscores the importance of tailored fall prevention strategies.

Implications

The findings highlight the critical role of nursing officers in preventing falls among pediatric patients and reinforce the importance of targeted educational interventions. By equipping nurses with specialized knowledge on child development, environmental risks, and parental involvement, healthcare institutions can enhance patient safety and reduce fall-related incidents. Fall prevention strategies should be tailored to children's unique needs, integrating age-appropriate interventions and continuous monitoring to improve outcomes.

Furthermore, this study identifies the barriers and facilitators that affect nurses' KAP of fall prevention. Understanding these factors can help guide the development of policies, training programs, and protocols to foster a culture of safety in pediatric healthcare settings. Future research should explore pediatric-specific fall prevention strategies and their implementation across diverse healthcare settings.

Limitations and future recommendations

Despite the study's significant findings, several limitations need to be addressed. The research was conducted in a single healthcare setting, which limits the generalizability of the results to other institutions with different staffing structures, patient populations, and fall prevention protocols. Future studies must adopt a multi-center approach to enhance the broader applicability of these findings. While the intervention effectively improved nurses’ KAP, this study did not assess long-term knowledge retention or sustained behavioral changes. Future research should incorporate follow-up assessments over extended periods to evaluate the durability of these improvements and determine whether periodic refresher training is needed. Another limitation is the reliance on self-reported data for attitudes, which may introduce social desirability bias. Future studies should implement objective measures, such as direct observations or electronic monitoring, to ensure a more accurate assessment of actual practice changes. Finally, pediatric fall prevention requires age-specific and condition-specific interventions, yet this study focused on general fall prevention measures. Future research must develop and evaluate tailored interventions that address the unique developmental and physiological characteristics of pediatric patients. These limitations highlight the need for ongoing professional development, stronger institutional support, and continued research to optimize pediatric fall prevention strategies.

## Conclusions

This study highlights the impact of structured educational interventions on nurses' KAP of pediatric fall prevention. While targeted training significantly improves safety awareness and clinical practices, sustaining these gains in daily nursing care remains a challenge. Falls among hospitalized children are largely preventable, yet persistent gaps in awareness, inconsistent adherence to protocols, and varying clinical environments contribute to ongoing risks. This highlights the need for ongoing education, regular monitoring, and strong institutional support to reinforce fall prevention strategies.

Pediatric patient safety must be a priority in all healthcare settings. Nurses, hospital administrators, and policymakers must work together to integrate fall prevention strategies into routine care and ensure that evidence-based practices become standard. Future efforts should focus on long-term knowledge retention, the effectiveness of different training methods, and the role of organizational culture in sustaining safety improvements. This study serves as a call to action. By advocating for structured educational programs, we can strengthen pediatric care, promote safer hospital environments, and take decisive steps toward preventing falls.
